# Genetic factors affecting storage and utilization of lipids during dormancy in *Mycobacterium tuberculosis*

**DOI:** 10.1128/mbio.03208-23

**Published:** 2024-01-18

**Authors:** Alexander Sturm, Penny Sun, Julian Avila-Pacheco, Anne E. Clatworthy, Zohar Bloom-Ackermann, Michael G. Wuo, James E. Gomez, Soomin Jin, Clary B. Clish, Laura L. Kiessling, Deborah T. Hung

**Affiliations:** 1 Infectious Disease and Microbiome Program, Broad Institute, Cambridge, Massachusetts, USA; 2 Department of Molecular Biology and Center for Computational and Integrative Biology, Massachusetts General Hospital, Boston, Massachusetts, USA; 3 Department of Genetics, Harvard Medical School, Boston, Massachusetts, USA; 4 Metabolomics Platform, Broad Institute, Cambridge, Massachusetts, USA; 5 Department of Chemistry, MIT, Cambridge, Massachusetts, USA; Washington University in St. Louis, St. Louis, Missouri, USA

**Keywords:** *Mycobacterium tuberculosis*, dormancy, lipid metabolism

## Abstract

*Mycobacterium tuberculosis* (Mtb) can adopt a non-growing dormant state during infection that may be critical to both active and latent tuberculosis. During dormancy, Mtb is widely tolerant toward antibiotics, a significant obstacle in current anti-tubercular drug regimens, and retains the ability to persist in its environment. We aimed to identify novel mechanisms that permit Mtb to survive dormancy in an *in vitro* carbon starvation model using transposon insertion sequencing and gene expression analysis. We identified a previously uncharacterized component of the lipid transport machinery, *omamC,* which was upregulated and required for survival during carbon starvation. We show that OmamC plays a role both in increasing fatty acid stores during growth in rich media and enhancing fatty acid utilization during starvation. Besides its involvement in lipid metabolism, OmamC levels affected the expression of the anti-anti-sigma factor *rv0516c* and other genes to improve Mtb survival during carbon starvation and increase its tolerance toward rifampicin, a first-line drug effective against non-growing Mtb. Importantly, we show that Mtb can be eradicated during carbon starvation, in an OmamC-dependent manner, by inhibiting lipid metabolism with the lipase inhibitor tetrahydrolipstatin. This work casts new light into the survival processes of non-replicating, drug-tolerant Mtb by identifying new proteins involved in lipid metabolism required for the survival of dormant bacteria and exposing a potential vulnerability that could be exploited for antibiotic discovery.

**IMPORTANCE:**

Tuberculosis is a global threat, with ~10 million yearly active cases. Many more people, however, live with “latent” infection, where *Mycobacterium tuberculosis* survives in a non-replicative form. When latent bacteria activate and regrow, they elicit immune responses and result in significant host damage. Replicating and non-growing bacilli can co-exist; however, non-growing bacteria are considerably less sensitive to antibiotics, thus complicating treatment by necessitating long treatment durations. Here, we sought to identify genes important for bacterial survival in this non-growing state using a carbon starvation model. We found that a previously uncharacterized gene, *omamC*, is involved in storing and utilizing fatty acids as bacteria transition between these two states. Importantly, inhibiting lipid metabolism using a lipase inhibitor eradicates non-growing bacteria. Thus, targeting lipid metabolism may be a viable strategy for treating the non-growing population in strategies to shorten treatment durations of tuberculosis.

## INTRODUCTION

Most antibiotics kill or inhibit the growth of actively dividing bacteria. However, many clinically relevant, pathogenic bacteria can adopt a non-growing state that is tolerant toward many antibiotics (1–7). Non-growing, dormant bacterial subpopulations (1) thus can constitute a considerable obstacle to effective antimicrobial therapy and contribute to persistent or recurring infections (8). *Mycobacterium tuberculosis* (Mtb), the causative agent of tuberculosis (TB), is one such important pathogen, wherein dormant subpopulations are thought to exist alongside growing bacteria during active infection (9) and underlie the need for a months-long multi-drug antibiotic regimen of which only rifampicin, among the drugs in the standard four-drug regimen, affects the dormant subpopulation (10). Since non-eradicated, dormant Mtb subpopulations can result in reactivation of latent infection or treatment failure of active TB, a better understanding of the biology of this subpopulation would inform the development of alternative antimicrobial strategies that more effectively eliminate dormant subpopulations.

While dormant bacteria are non-growing, they are not metabolically inert. A residual metabolism must be maintained to provide electron equivalents for the polarization of the cell membrane to produce ATP (11, 12) and to allow repair and replacement of damaged cell components. While much remains to be understood about what distinguishes the metabolism of dormant, non-growing vs actively growing Mtb *in vivo,* multiple studies have demonstrated that the bacteria have a unique ability to assimilate and utilize host lipids [in particular fatty acids and cholesterol (13–15)], which are important carbon sources facilitating the bacterium’s survival. In addition, lipid-loaded macrophages, termed foamy macrophages, play a critical role in Mtb survival in the granuloma (16, 17). The importance of lipid metabolism is also reflected in the large number of Mtb genes (250) devoted to lipid metabolism in comparison to *Escherichia coli* (50) (18) and the extraordinarily high concentration of lipids (up to 60%) in Mtb’s dry weight (19) in comparison to *E.coli* or eukaryotic cells [10%; (20)]. It is unknown how dormant Mtb balances its utilization of available environmental lipids such as those obtained from the host vs its own readily available lipids. Nevertheless, *in vitro,* carbon starvation models and *in vivo* chronic mouse infections demonstrate, albeit indirectly, that fatty acids are an essential carbon source during dormancy. Mtb growth using fatty acids as the sole carbon source requires the malate synthase *glcB* and the glyoxylate shunt consisting of isocitrate lyases *aceA* and *icl1* (21–25). Deleting the latter gene, *icl1,* diminishes Mtb survival during carbon starvation in activated macrophages and chronic mouse infections (21, 23, 26). These data thus indirectly support that fatty acids are an essential carbon source *in vivo* during dormancy.

The structure and composition of the mycobacterial cell envelope are unique among bacteria, with the outermost mycomembrane layer composed of an inner leaflet of long hydrophobic mycolic acids and an outer leaflet of non-covalently bound lipids and glycolipids. While Mtb encodes a phalanx of lipid transport systems that support the organization of this unique cell envelope, specific knowledge about its construction, maintenance, and lipid transport is still limited (27). Still, these systems are critical for infection of mice (28). Four operons encoding lipid transport systems (*mce1-4*), originally named for mammalian cell entry (Mce) proteins (29), are distributed across the genome. The Mce transporters share a similar structural organization, consisting of six *mce* proteins and two permeases that assemble, along with an ATPase, to form an ABC transporter believed to reside in the extracellular membrane. Moreover, three of the four operons encode pairs of *mce*-associated (*mam*) genes, with Mams thought to stabilize Mce transporters (30). Finally, there are five additional orphaned *mam* genes (*omamA-E*) that are unlinked to the *mce* operons with low level of homology to the *mam* genes encoded in the *mce* clusters (31).

Among the Mce transporters, Mce4 is best understood. It transports cholesterol across the membranes, is essential for Mtb growth on sterols as a sole carbon source, and is thought to have a role in pathogenicity as *mce4* deletion mutants are attenuated in mice (31–34). Similarly, Mce1 has been shown to be a fatty acid transporter in Mtb (35), affecting a variety of phenotypes including the concentration of mycolic acids (36), the immune response (37), development of granulomas in mice (37), and importantly, reactivation of mouse infections post-antibiotic treatment (38). Due to their homology with each other and the abundance of orphaned *mam* genes encoding Omam proteins, the components of the different Mce transporters are thought to interact and be involved in cross talk. For example, it has been reported that the orphaned Mce accessory protein Rv0199/OmamA plays a role in cholesterol utilization, which is an Mce4-dependent process, and in stabilizing the Mce1 transporter complex (30). This cross talk, initially observed for one orphaned Mam (OmamA), likely extends to the other four *omam* genes and suggests possible redundancy in their interaction with various clusters, likely depending on environmental cues (30).

To better understand the biology of Mtb survival of dormancy, we explored the genetic requirements and transcriptome shifts during carbon starvation and subsequent resuscitation *in vitro*. Carbon starvation is one of the stresses encountered inside the phagosomes of infected macrophages (39, 40) and, therefore, serves as a model for non-growing subpopulations during active or latent disease. We found that components of the lipid transport systems comprised *mce* and *mce*-associated genes play a role in Mtb survival during carbon starvation using a transposon mutant library and transposon insertion sequencing. Furthermore, we show that one of these genes, the orphaned *mce*-associated gene *omamC*, which is highly expressed during these conditions, allows Mtb to store more lipids during growth and metabolize fatty acids more rapidly during carbon starvation. Our findings underscore that dormant subpopulations of Mtb can be effectively eliminated by inhibiting lipid hydrolysis, achieved by treatment with the lipid esterase inhibitor terahydrolipstatin (THL) in an OmamC-dependent manner. This work supports the notion that lipid hydrolysis is vital to the survival of Mtb during carbon starvation and adds to the growing recognition of the importance of lipid metabolism during dormancy as a target in antibiotic-tolerant non-growing Mtb.

## RESULTS

### A genetic screen reveals an uncharacterized lipid transport gene required for and upregulated during dormancy

**Fig 1:**
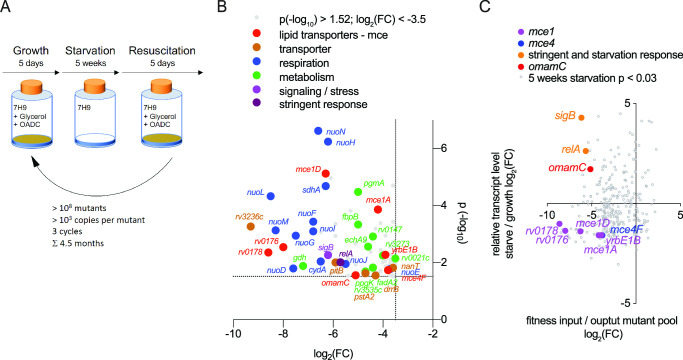
Tn-seq and RNAtag-seq reveal that *omamC* is critical for survival during carbon starvation. (**A**) Carbon starvation-resuscitation growth scheme. A pooled *M. tuberculosis* H37Rv transposon library was grown in rich media [7H9, 10% oleic acid-albumin-dextrose-catalase (OADC), 0.5% glycerol, 0.05% tyloxapol] for 5 days, diluted into starvation media (7H9 salts, 0.05% tyloxapol), and cultured for 5 weeks, followed by resuscitation in rich media for 5 days. Transposon mutants present in the pool prior to carbon starvation and resuscitation cycling (input pool) and after three cycles of growth, starvation, and resuscitation performed over 4.5 months (output pool) were determined using Tn-seq. (**B**) Gene set enrichment analysis of genes (102) that were significantly [*P* < 0.03 (Mann-Whitney *U* test); log_2_(FC) <−3.5] underrepresented in the output pool after three cycles of starvation and resuscitation. Genes are color coded based on the pathway with which they are associated with the positive control genes *sigB* and *relA*, known to be involved in carbon starvation, indicated in purple (compare Fig. S4; Table S3). (**C**) Of seven *mce* genes defined as hits by Tn-seq (**B**), only *omamC* is expressed during carbon starvation. Depicted are relative transcript levels during starvation of 298 genes whose corresponding transposon mutants were significantly changed in the starvation-resuscitation Tn-seq screen (with a threshold of *P* < 0.03, Mann-Whitney U test (MWU)). Differences in gene expression during carbon starvation compared to logarithmic growth were determined using RNAtag-seq and DESeq2 (R). The results of the Tn-seq screen are shown as fitness, i.e.*,* a function of the transposon input/output pool. The log_2_(FC) is shown in both cases. Depicted in gray are all genes that had a *P*-value <0.03 (*P* (-log_10_) >1.52) in the Tn-seq analysis. Carbon starvation controls *sigB* and *relA* (48, 49) are shown in purple. All values are listed in Tables S4 to S6.

*In vitro*, carbon starvation has been used to model dormancy in Mtb (41, 42), where the bacterium can survive months of carbon starvation with minimal loss of viability in comparison to other bacterial species (Fig. S1). To better understand which Mtb genes are required to survive dormancy, we screened a transposon mutant library of >40,000 mutants created with the phiMycoMarT7 element (43),(44) for mutants that were impaired for survival during carbon starvation (39, 41). We subjected the transposon mutant pool *in vitro* to carbon starving for 5 weeks followed by a 5-day resuscitation period in growth media and repeated three cycles of growth, starvation, and resuscitation over a total of 4.5 months (Fig. 1A). The cycling had the advantage that differences in the numbers between survivor and non- or impaired survivor mutants would amplify. To avoid bottlenecks created by cycling the transposon mutant pool, we ensured a passage of more than 10^8^ cells during each step of the experiment (on average >1,000× mutants per TA site) and determined the copy number for each transposon mutant before and after carbon starvation using transposon insertion sequencing (Tn-seq) (34, 42, 45, 46). We identified mutants significantly underrepresented in the output pool compared to the input pool using the previously developed conditional essential genes identifier con-ARTIST (47). Mutants of interest were defined as having a significance value of *P* < 0.03 (Mann-Whitney *U* test) and a log 2-fold-change [log_2_(FC)] between input and output pools of <−3.5. This methodology was highly reproducible in two replicates of the experiment (*r*^2^ = 0.78, Fig. S2; Table S1).

We identified 102 genes and 6 intergenic regions that were significantly underrepresented in the output pool compared to the input pool, indicating that these mutations resulted in a high fitness cost to the bacterium during carbon starvation and/or resuscitation (Fig. S3; Table S2). Gene set enrichment analysis (GSEA) revealed that mutations that conferred the most substantial fitness cost were those in genes involved in oxidative phosphorylation [*nuo* genes, *sdh*, *cydA*; normalized enrichment score (NES) = −2.2], lipid transporters (*mce* genes, NES = −1.5), the dormancy regulator *sigB* (Fig. 1B; Fig. S4), other transporters, and several genes involved in different metabolic pathways. In addition, the stringent response regulator *relA* was among the top hits, thus corroborating the validity of our results (48, 49). Of note, the toxin-anti-toxin systems that are often referred to as being crucial for establishing persistent or dormant populations were not among the hits, which is perhaps attributable to functional redundancy among the 88 toxin-anti-toxin systems in Mtb (50).

**Fig 2:**
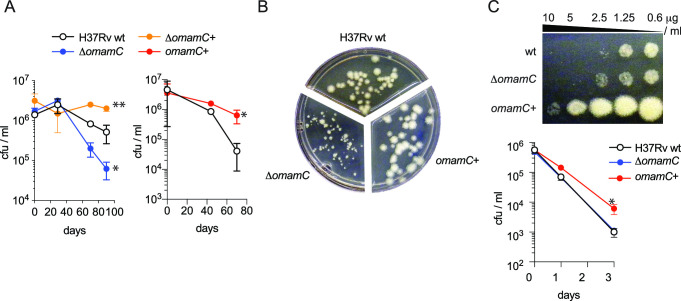
*omamC* is important for survival during carbon starvation and rifampicin treatment. (**A**) Deletion of *omamC* is detrimental to survival, while overexpression of *omamC* improves survival during carbon starvation. H37Rv wild type, an *omamC* deletion strain (Δ*omamC*), the deletion strain complemented with *omamC* (Δ*omamC+*), and a strain overexpressing *omamC* (*omamC*+) were carbon starved 80–100 days. Culture viability was determined by quantifying the number of colony-forming units (cfu) on growth-permissive solid agar plates over time (mean ± SD; *, *P*_Δ*omamC*_ = 0.035; **, *P*_Δ*omamC*+_ = 0.009; *, *P_omamC_*_+_ = 0.028, two-tailed non-parametric *t*-test). (**B**) *OmamC* overexpression (*omamC*+) increases colony size, while *omamC* deletion (Δ*omamC*) decreases colony size compared to wild type (H37Rv) when 5-week carbon-starved cultures are plated to 7H10 plates (all cultures were normalized to OD_600_ prior to carbon starvation entry). (**C**) Overexpression of *omamC* (*omamC*+) increases the minimal bactericidal concentration of rifampicin compared to wild type or Δ*omamC* during carbon starvation. Carbon-starved strains were exposed to rifampicin over 3 days and then spotted onto solid agar plates without rifampicin (upper panel), or the viability of rifampicin-exposed cultures was determined over time by quantifying the number of cfu on solid agar (lower panel, 20 ug/mL rifampicin; mean ± SD; *, *P*_Δ*omamC*+_ = 0.0178, two-tailed non-parametric *t*-test).

The identification of the importance of *mce* lipid transporters for survival during carbon starvation raised the hypothesis that lipids could serve as a vital energy source during dormancy. Among the four *mce* gene clusters present in Mtb (51, 52), we identified five genes in the *mce1* cluster, one in the *mce4* cluster, and the uncharacterized orphaned *mce* gene *omamC* as being important for survival during carbon starvation (30) (Fig. 1B; Fig. S5). Since the Tn-seq screen did not distinguish between genes required for survival during carbon starvation and genes required for resuscitation, we performed a transcriptomic analysis comparing a 5-week carbon-starved wild-type culture with a culture that had been subsequently resuscitated (Table S3). Among the seven *mce* genes identified in the Tn-seq data set, *omamC* had mRNA levels uniquely enriched during carbon starvation compared to growth in rich media [differential expression analysis (DESeq2), log_2_(FC) = 1.7, Fig. 1C]. As expected, the stress regulators *sigB* and *relA* were also upregulated during starvation [log_2_(FC) = 2.6 and 4.3, respectively, Fig. S6]. Although the absence of changes in the transcript levels for other *mce* genes does not exclude their involvement in carbon starvation, we focused on understanding how the poorly characterized, orphaned *mce* gene *omamC* affects Mtb survival during carbon starvation.

### OmamC plays a role in fitness during dormancy and in rifampicin tolerance

We constructed a clean deletion of the *omamC* gene in the Mtb strain H37Rv (Δ*omamC*). We confirmed that, indeed, deletion of *omamC* results in a moderate but significant reduction in Mtb’s ability to survive carbon starvation (Fig. 2A). These findings are similar to previous observations with major dormancy regulators like *relA* (52). Meanwhile, constitutive overexpression of *omamC*, in either the wild-type or Δ*omamC* background, rendered Mtb more resilient to carbon starvation stress (two-tailed non-parametric *t*-test *P*_Δ*omamC*_ = 0.035, *P*_Δ*omamC−*_ *P_omamC_*_+_ = 0.009, Fig. 2A), an observation also supported by a calcein-based viability assay (Fig. S7). Perturbing *omamC* during carbon starvation and resuscitation on solid agar also affected colony size, with deletion of *omamC* resulting in the formation of smaller colonies, while overexpression (*omamC*+) resulted in larger colonies (Fig. 2B). While OmamC-dependent differences in colony size on solid agar could arise from differences in growth rate, no *omamC*-dependent differences in liquid axenic culture were observed (two-tailed non-parametric *t*-test, *P* = 0.01, Fig. S8A). However, *omamC*+ was slightly impaired in its ability to actively replicate inside J774 macrophages compared to wild-type Mtb (Fig. S8B).

Since *in vitro* carbon starvation models have been shown to induce a drug-tolerant state that is refractory to killing by the majority of anti-tubercular agents, with the exception of rifampicin (10), we examined whether perturbations in the ability to survive carbon starvation might affect the efficacy of rifampicin. Overexpression of *omamC* increased tolerance toward rifampicin as reflected in an increase of the minimal bactericidal concentration and a decreased kill rate of pre-starved Mtb (two-tailed non-parametric *t*-test, *P* = 0.0178; Fig. 2C) (50).

### OmamC increases Mtb fatty acid stores during growth and utilization during starvation

We hypothesized that *omamC* could be involved in lipid metabolism, given its modest similarity with two other *mce*-associated genes, *rv0177* and *rv1972* (Fig. S9). While Mce-associated proteins are poorly characterized (53), their interaction with Mce membrane complexes (35, 54) suggests that they could play some role in conferring lipid substrate specificity. We thus considered two possible roles for OmamC that might contribute to better survival during carbon starvation: (i) OmamC could increase the uptake or biosynthesis of (certain) fatty acids during logarithmic growth in nutritional replete environments so that Mtb has increased stores of accessible fatty acids going into carbon starvation, and/or (ii) OmamC could affect fatty acid oxidation during carbon starvation to increase fatty acid utilization and thus improve the bacterium’s survival of carbon starvation. In this carbon starvation model, since fatty acids are not available from the environment through nutrient supplementation, Mtb would only be able to utilize its own stored lipids or scavenge lipids from deceased siblings.

**Fig 3:**
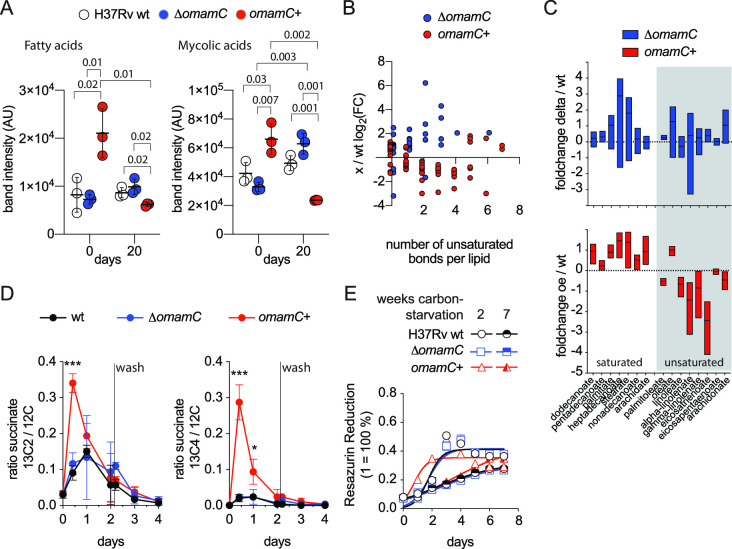
OmamC affects lipid quantity and composition in bacterial cells during growth in rich media and starvation. (**A**) *OmamC* overexpression (*omamC*+, red circles) significantly increased the fatty acid (left panel) and mycolic acid (right panel) content prior to carbon starvation compared to wild type (H37Rv, open circles) or an *omamC* deletion (Δ*omamC*, blue circles), while after 20 days of carbon starvation, this observation was reversed with *omamC*+ displaying a modest but significant reduction in both fatty (left panel) and mycolic acids (right panel) compared to wild type. Strains were grown in rich media supplemented with [1 C^14^] acetate to label the lipid pool and then switched to carbon starvation media. C^14^-labeled fatty and mycolic acids were quantified at indicated time points by thin layer chromatography. Data shown, mean ± SD; *P*-values (unpaired *t*-test). (**B**) Overexpression of *omamC* (*omamC*+) results in a lower concentration of unsaturated fatty acids relative to wild-type H37Rv in 3 weeks of starved cultures. Conversely, deletion of *omamC* (Δ*omamC*) results in higher concentrations of unsaturated fatty acids relative to wild-type H37Rv. Relative concentrations of lipids (mono-, di-, tri-acyl glycerides, and other fatty acid esters, see Tables S11 and S12) were quantified by liquid chromatography-mass spectrometric (LC-MS) during starvation. Lipids from Δ*omamC* or *omamC*+, whose amounts were significantly different from that in wild type, are plotted according to their number of unsaturated bonds per lipid (*x*-axis), shown in log_2_(FC) (biological triplicates, *P* < 0.05, two-tailed non-parametric *t*-test). (**C**) Overexpression of *omamC* (*omamC*+) resulted in an enrichment of saturated free fatty acids and a reduction in unsaturated free fatty acids relative to wild-type H37Rv (bottom panel), while deletion of *omamC* (Δ*omamC*) had a less pronounced effect when compared to wild-type H37Rv (top panel). Relative concentration of selected free saturated (left group of fatty acids on the *x*-axis) and unsaturated (right group of fatty acids on the *x*-axis, gray area) fatty acids during starvation as determined by LC-MS between Δ*omamC* and wt (blue), and *omamC*+ and wild type (red). (**D**) *omamC+* facilitates the early, rapid incorporation of [all-C^13^]-oleic acid into the TCA cycle intermediate succinate following 3 weeks of starvation. Succinate concentrations in wild type (black), Δ*omamC* (blue), and *omamC*+ (red) were determined by LC-MS following the addition of [all-C^13^]-oleic acid to carbon-starved cells. Incorporation of C^13^ into succinate is shown as a ratio of succinate molecules containing 2 C^13^ (left panel; ***P_omamC__+ vs WT_ = 0.0002, two-tailed unpaired *t*-test) or 4 C^13^ (right panel; ***P_omamC__+ vs WT_ = 0.0008; *P_omamC__+ vs WT_ = 0.0271; two-tailed unpaired *t*-test) atoms compared to those consisting of only C^12^ atoms, of which the majority originate from before the addition of [all-C^13^]-oleic acid. (**E**) Following 2 weeks of carbon starvation, *omamC+* (red) was able to rapidly metabolize the unsaturated fatty acid oleic acid more efficiently than wild type or Δ*omamC* (blue), an effect that vanished after 7 weeks of starvation without remarkable differences between the three strains. Metabolic activity of the culture was assayed daily for over 1 week by measuring the reduction of the redox indicator resazurin. The experiment was performed in sextuplicate, and error bars indicate standard deviation.

To explore whether OmamC might play a role in fatty acid storage in times of carbon availability and utilization during periods of carbon starvation, we compared the incorporation of [1-^14^C] acetate into the lipids of mutants in which *omamC* was deleted or overexpressed (Δ*omamC* and *omamC*+, respectively). We grew Mtb in the presence of [1-^14^C] acetate 1 day (starting at day −1) before we subjected cultures to carbon starvation and measured the concentration of fatty and mycolic acids using thin layer chromatography (TLC) at day 1 and 20. On day 1 after exposure to [1-^14^C] acetate in rich media, *omamC+* had twice the amount of ^14^C-labeled fatty acids and 1.5 times the amount of ^14^C-labeled mycolic acids compared to wild type (normalized to cell number, Fig. 3), suggesting that OmamC plays a role in increasing uptake of [1-^14^C] acetate and its incorporation into fatty and mycolic acids during periods of exposure to a rich environment. While we did not observe statistically significant alterations in ^14^C-labeled incorporation into fatty acids in Δ*omamC*, we did observe subtle trends that are consistent with this role for OmamC. The lack of a more robust phenotype for Δ*omamC* could be attributed to some functional redundancies between *mam* genes, with OmamC’s role in these phenotypes being more pronounced following overexpression rather than deletion.

After 20 days of carbon starvation, the picture had reversed, and *omamC*+ had a modest but statistically significant lower amount of ^14^C-labeled fatty and mycolic acids compared to wild type (*omamC*+ day 1 vs day 20: *P*_fatty acids_ = 0.01; *P*_mycolic acids_ = 0.002), while Δ*omamC* had significantly more ^14^C-labeled mycolic acid compared to wild type (Δ*omamC* day 1 vs day 20: *P*_mycolic acids_ = 0.003, Fig. 3A). These findings are consistent with a role for OmamC in enhancing fatty and mycolic acid utilization.

Apart from alterations in the absolute concentration of fatty and mycolic acids during carbon starvation, the composition of lipids was also affected by OmamC. Using liquid chromatography-mass spectrometric (LC-MS) analysis, compared with wild type, we found that during starvation, *omamC*+ had a relatively lower concentration of lipids containing unsaturated fatty acids (Fig. 3B; Tables S4 and S5). This observation was also reflected in the pool of free fatty acids for *omamC*+, where unsaturated fatty acids were underrepresented and saturated fatty acids overrepresented compared to wild type (Fig. 3C). Meanwhile, the opposite result was observed for Δ*omamC*, with a relative increase in lipids with unsaturated fatty acids compared to wild type (Fig. 3B and C).

These data show that prior to dormancy entry, in a setting of rich media, overexpression of *omamC* results in an enrichment of fatty and mycolic acids during nutrient-rich periods, which are then able to be utilized during periods of starvation, with preferential utilization of unsaturated fatty acids. In contrast, while we did not observe alterations in the Δ*omamC’s* ability to store fatty or mycolic acids during periods of growth in rich media, Δ*omamC* was unable to utilize available mycolic acids and unsaturated fatty acids as effectively as wild type or OmamC+ during periods of starvation. Taken together, these data suggest that OmamC plays a role in lipid storage during growth in rich media and lipid utilization during starvation.

Finally, to examine OmamC’s role in fatty acid oxidation and the utilization of fatty acids for central metabolism during starvation, we used LC-MS to measure the incorporation of [all-^13^C]-oleic acid into succinate, a key metabolite in the TCA cycle. We starved cells for 3 weeks, then added [all-^13^C]-oleate to cells in order to compare the incorporation of ^13^C into succinate in wild type, Δ*omamC,* and *omamC*+. In all cases, we observed an initial burst of ^13^C incorporation into newly produced succinate 10 hours after the addition of [all-^13^C]-oleic acid. However, we measured a 3-to-14-fold increase in isotopically labeled succinate (two or four ^13^C atoms incorporated per molecule succinate, Fig. 3D) in *omamC*+ compared to wild type or Δ*omamC*. These findings were similarly supported by the finding that in response to supplementation with oleic acid as a sole carbon source, carbon-starved *omamC*+ metabolically responded, as measured with the redox-sensitive dye resazurin, more rapidly than wild type or Δ*omamC* after 2 weeks of starvation (Fig. 3E). In general, adding fatty acids with various degrees of saturation also triggered a faster response of *omamC*+ (Fig. S10). After 7 weeks of carbon starvation, however, there was little difference in the ability of these strains to metabolize oleic acids or other fatty acids (Fig. 3E; Fig. S10).

### OmamC regulates α-α-sigD and other genes that increase fitness in carbon starvation

**Fig 4:**
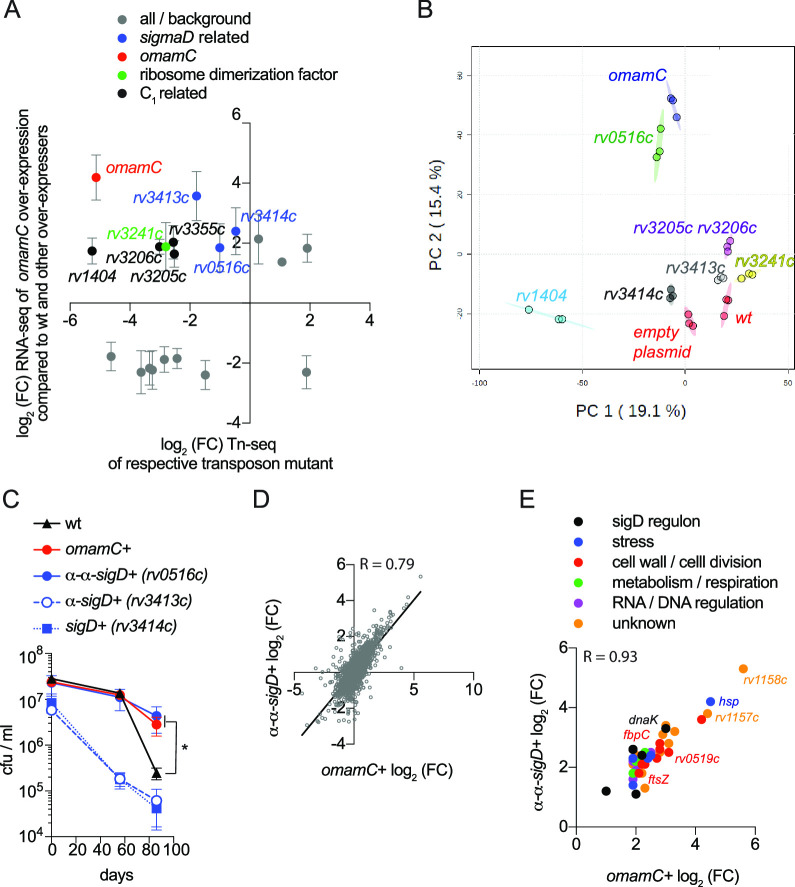
*omamC*+’s ability to survive carbon starvation correlates with *α-α sigD* expression and an array of genes under the regulation of *omamC* and *α-α-sigD*. (**A**) Differential expression analysis (R) of genes upregulated in *omamC*+ compared to wild-type H37Rv and four other control strains overexpressing genes with no fitness benefit during carbon starvation (*rv0246+*, *rv1374c+*, *rv2633c*+, and *rv2710+*, Fig. S15). Genes significantly differentially expressed [log_2_(FC) >1.2 or <−1.2] in *OmamC*+ compared to all five reference strains are shown (*y*-axis, gray, Tables S7 and S8), plotted against each gene’s fitness impact from the original transposon screen (Fig. 1B; Tables S4 and S5). Error bars represent the standard deviation between differential expression values among the five reference strains. Genes were colored according to their functional annotations as follows: *sigD* regulon, blue; C_1_ metabolism or regulation (methyltransferases, pterin cofactor regulation), black; and ribosome dimerization factor, green. (**B**) Genes identified as overexpressed in *omamC*+ (**A**) were overexpressed in H37Rv, and the expression profiles of these strains, along with H37Rv and *omamC*+, were subjected to principal component analysis following 3 weeks of carbon starvation (Table S8). Only the α-α *sigD (rv0516c*) overexpressing strain (α-α *sigD+*) clustered with *omamC+,* while the expression profiles of the remaining six overexpressing strains clustered with wild-type H37Rv. (**C**) Overexpression of α-α-*sigD* (α-α-*sigD+, rv0516c*) phenocopied the ability of *omamC*+ to survive carbon starvation compared to H37Rv and strains overexpressing *sigD* or α-*sigD*. Survival during carbon starvation was assayed as described in Fig. 2. Error bars indicate the standard deviation among triplicates (*P_α-α-sigD+__vs WT_ = 0.0482; *P_omamC__+ vs WT_ = 0.0237; two-tailed unpaired *t*-test). (**D**) The expression profiles of 3-week carbon-starved *omamC+* and α-α *sigD+* (*rv0516c+*) [normalized to carbon-starved wild-type cells (DESeq2), tightly correlated (*R* = 0.79). (**E**) The top 30 most upregulated genes in both *omamC*+ and α-α-*sigD*+, color coded by function (Table S10), correlated tightly (*R* = 0.93).

The *omamC* gene is in an operon encoding putative stress regulators (*rv1363c*; Fig. S13). This location contrasts with that of other *mce*-associated genes, which are usually encoded in gene clusters associated with Mce transporters and are known to be important for shifting lipid composition, cholesterol metabolism (*mce4* cluster), and infection. Given its atypical genomic association, we explored what other pathways might be impacted by overexpression of *omamC*. We compared the expression profiles of *omamC*+ to a set of five strains, including wild type and four control strains overexpressing genes with no fitness consequence during carbon starvation (Fig. S12). Differential expression analysis (Tables S6 and S7) revealed that only eight genes were upregulated in *omamC*+ compared to the five reference strains after 3 weeks of carbon starvation [log_2_(FC) >1.2]. *OmamC* transcripts themselves were eight times enriched. Interestingly, the transposon mutants corresponding to all eight genes had also shown a fitness defect in the initial Tn-seq experiment (Table S2) and were either related to the stress regulon sigma D (*rv0516c, rv3413c, rv3414c*), molybdopterin metabolism/regulation (*rv1404, rv3205c, rv3206c, rv3355c*) or ribosome dimerization (*rv3241c*, Fig. 4). Seven of the eight genes (except *rv3355c*) showed enriched transcript levels during starvation, which decreased during resuscitation in the wild-type background, a pattern similar to that observed for *omamC* expression (Fig. S13).

We were interested if any of these genes when overexpressed results in similar expression profiles to *omamC*+ using a principal component analysis (Table S8) to define components of a more general transcriptome supportive of carbon starvation survival. Indeed, the profile of the α-α *sigD (rv0516c*) overexpressing strain (α-α *sigD+*) clustered with *omamC+*, while all other expression profiles clustered with wild type (Fig. 4B). Surprisingly, the *sigD (rv3414c*) overexpression strain (*sigD+*) fell in the cluster with wild type, suggesting that the effects of α-α *sigD (rv0516c*) overexpression might not be mediated by the *sigD* regulon. In addition to the similarity between the transcriptomes of *omamC* and α-α *sigD+,* α-α *sigD+* also phenocopied *omamC*+ in its enhanced ability to survive carbon starvation compared to wild type or strains overexpressing *sigD* or *α-sigD,* where overexpression of the latter two genes was detrimental to survival (Fig. 4C).

Since the expression profiles of carbon-starved α-α *sigD+* and *omamC*+ correlated well (*R* = 0.79, both transcriptomes were normalized to carbon-starved wt using DESeq2, Fig. 4D; Table S8) and both displayed an enhanced ability to survive carbon starvation, we speculated that genes that are upregulated in these two strains might be important during dormancy. The top 30 most upregulated genes included three members of the *sigD* regulon [*dnaK*, an hsp70 co-factor, and a thiol peroxidase (Fig. 4E; Fig. S14; Tables S9 and S10)] (55), five genes involved in stress responses, and eight genes involved in cell wall maintenance and cell division, including *fbpC* and *ftsZ*. The remaining half of the 30 most upregulated genes were either conserved hypothetical proteins, possible or probable transcriptional regulatory proteins, or were otherwise poorly annotated. Upregulation of *fbpC* was intriguing as it encodes one of the mycolyltransferases Ag85 that catalyzes the transfer of mycolic acids between trehaloses, consistent with our earlier finding that *omamC* overexpression results in increased consumption of fatty and mycolic acids during starvation. This finding suggests that there may be significant changes to the mycomembrane (Fig. 3) and mycolic acid metabolism, and its transcriptional upregulation may represent a compensatory mechanism for dwindling mycolic acid concentrations. The upregulation of *ftsZ*, involved in cell division, was also interesting, given the increased exit rate from dormancy in *omamC+*. We chose 5 from the top 30 highest expressed genes and constructed overexpression plasmids (*rv0251c, rv0519c, rv1158c, rv3084*). However, none conferred a survival advantage on their own during carbon starvation in the manner of *omamC+* and α-α *sigD+*, suggesting that either other genes are involved, or the concerted action of several genes under the impact of *omamC+* and α-α *sigD+* may be required to increase fitness (Fig. S15).

### OmamC defers killing by lipase inhibitor THL

**Fig 5:**
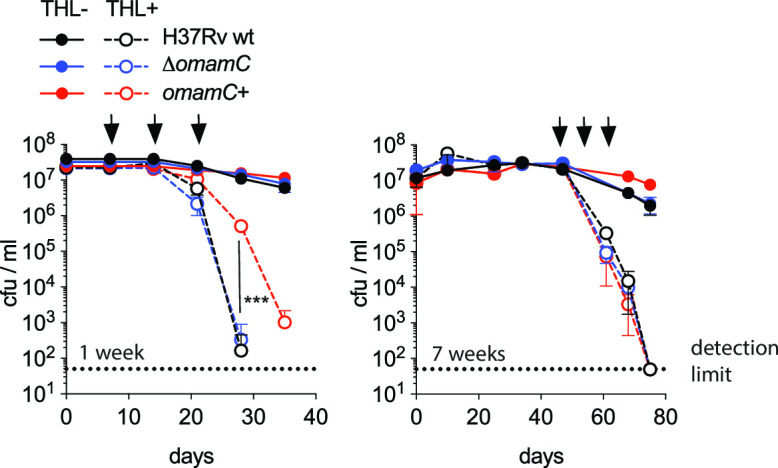
Lipid consumption is required for survival during carbon starvation, and *omamC* can contribute to this effect. The lipase and mycolyltransferase inhibitor THL was added to cultures (H37Rv, black; Δ*omamC*, blue; and *omamC*+, red) after 1 week (left panel) or 7 weeks (right panel) of carbon starvation at the indicated time points (black arrows, dashed lines), compared to untreated strains (solid lines). Culture viability was determined by quantifying the number of colony-forming units (cfu) in triplicate on growth-permissive solid agar plates over time. Error bars indicate the standard deviation; ***P_omamC__+ vs WT_ = 0.0003; two-tailed unpaired *t*-test).

Having identified a role for OmamC in lipid metabolism and survival during starvation, we sought to link the role of fatty acid utilization and survival during carbon starvation. We treated carbon-starved Mtb with tetrahydrolipstatin (THL), a non-specific lipid esterase inhibitor, including lipases and mycolyltransferases ( Fig. S16) (56). We reasoned this exposure would inhibit the release of free fatty acids for β-oxidation during starvation, thus hindering ATP production through lipid metabolism. THL treatment, indeed, resulted in Mtb killing (55–57), dramatically decreasing Mtb survival during carbon starvation at both early (1 week) and late (7 weeks) time points during starvation. Bacteria died within the course of a few days after THL exposure (Fig. 5). Of note, this killing was in an OmamC-dependent manner at the earlier time point as the expression of higher levels of OmamC allowed Mtb to delay the lethal effects of lipase inhibition. We did not observe the positive benefit of *omamC* overexpression when we added THL late, after 40 days, with all strains dying with similar kinetics. Overexpression of *omamC* also did not affect the minimal inhibitory concentration of THL in rich media ( Fig. S17). These data show that THL-dependent interference with lipid metabolism, potentially through inhibition of lipid consumption, is detrimental during dormancy and that the activity of OmamC can contribute to better survival in the early stages of dormancy.

## DISCUSSION

Adapting to a complex stress such as carbon starvation or conditions inside a macrophage requires many factors to support short- and long-term survival. In the context of Mtb infection, non-growing, dormant bacteria are tolerant to most antibiotics and may complicate TB treatment if this population is not eradicated and exits dormancy following treatment cessation to cause active disease. Therapeutic interventions that might target this non-growing subpopulation of Mtb could decrease the duration and increase the effectiveness of TB therapy. While some metabolic characteristics of Mtb during dormancy are coming into focus, there is still an incomplete understanding of this state. Metabolism in Mtb is slowed during dormancy but not abolished as is evidenced by gene expression levels that decrease yet do not disappear (58–60).

**Fig 6:**
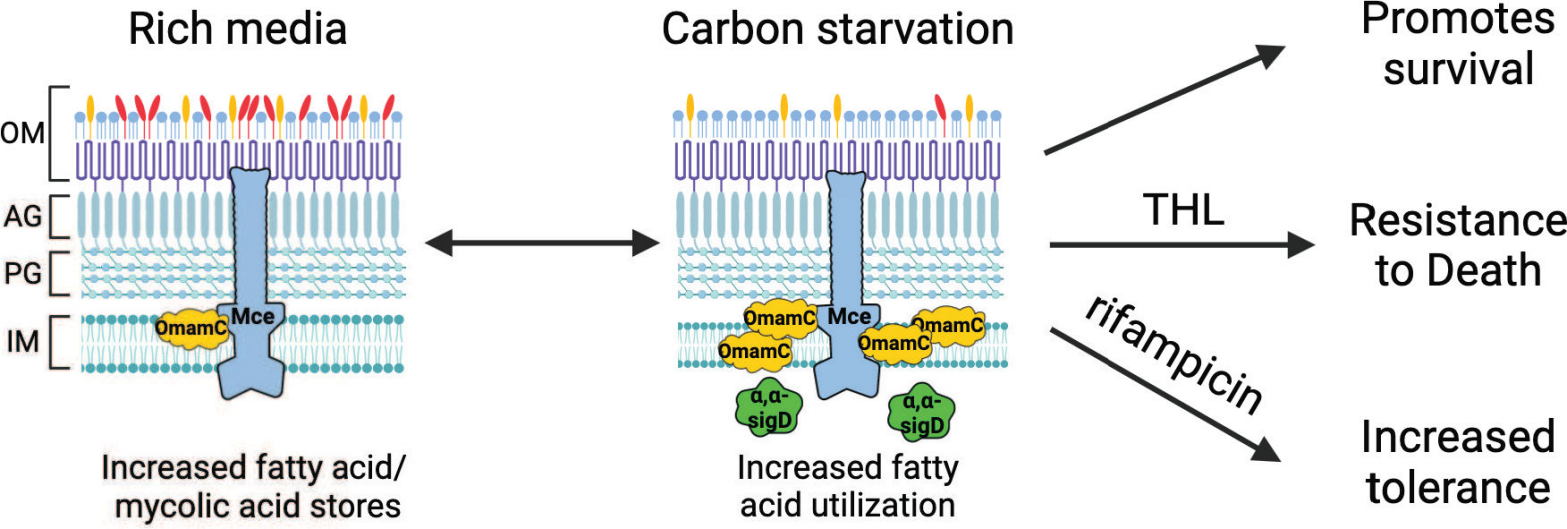
Possible model of OmamC activity during growth in rich media and carbon starvation. *OmamC* promotes increased storage of fatty and mycolic acids during growth in rich media and increased utilization of these stores in carbon starvation, which is associated with enhanced survival during carbon starvation, enhanced resistance to THL-mediated killing, and increased tolerance to rifampicin. *OmamC* is upregulated during starvation and promotes upregulation of α-α *sigD*, which acts in a non-canonical fashion that does not involve the *sigD* regulon to promote survival during carbon starvation. OM, outer mycolic acid layer; AG, arabinogalactan; PG, peptidoglycan; IM, inner membrane.

Within this context, we have now performed a genome-wide negative selection study to identify genes required for survival in a carbon starvation model of dormancy, with genes involved in oxidative phosphorylation and lipid transporters, including genes in the *mce1* and *mce4* clusters, emerging as critical. We have also identified *omamC*, a previously uncharacterized orphaned *mce* gene, as being required for survival during carbon starvation. We find that overexpression of OmamC results in both increasing lipid stores in rich media prior to starvation, which may account for changes in colony size, and more rapid utilization of lipids, particularly unsaturated fatty acids, during periods of starvation, which correlates with increased fitness of Mtb during dormancy (Fig. 6). We also find that α-α *sigD*, whose expression is regulated by OmamC expression, also impacts survival in dormancy, although in a non-canonical fashion that does not involve the *sigD* regulon. Importantly, when access to free fatty acids is limited by lipase and mycolyltransferase inhibition with THL, non-growing Mtb cannot survive and are eradicated, with early lipid utilization during dormancy being OmamC dependent. As Mtb uses lipids as a nutrient source during dormancy, interfering with fatty acid availability could be a viable strategy to eliminate non-growing Mtb, informed by the relative balance between a bacterium’s use of its own lipids and scavenging of external lipids during dormancy. These findings are consistent with reports from *E. coli* and *Salmonella* Typhimurium in which the *fad* regulon, involved in long-chain fatty acid transport, activation, and β-oxidation, can contribute significantly to their fitness during carbon starvation (61, 62).

Numerous publications have shown that Mtb requires lipids as a carbon source to survive in macrophages and to establish and maintain a successful infection in mice (23, 24, 35, 63–65). While the acquisition of lipids is less well understood, four Mce lipid transport systems in Mtb have been studied (31). Of these, only the function of Mce1 as a lipid transporter (35) and Mce4 as a sterol transporter has been identified (32, 34). Their importance in virulence and survival in macrophages has been shown in different infection models (31, 36, 37, 66, 67). However, none of these transport systems was shown to be explicitly important during carbon starvation or dormancy.

Beyond the transport proteins themselves, less attention has been paid to *mce*-associated genes (*mam*) co-encoded in the *mce* clusters or orphaned elsewhere in the genome (30, 51, 68). One of these genes, *omamA*, is required for cholesterol uptake with OmamA stabilizing the Mce4 complex as well as the Mce1 complex in macrophages (30, 68). We now show that OmamC is important during starvation, related to its role in lipid utilization. It is possible that OmamC could also physically or functionally interact with Mce1 since genes in this complex mirrored the OmamC mutant with substantial fitness costs during carbon starvation (Fig. 1), and *ΔomamC* phenocopies a Δ*mce1* mutant with increased free mycolic acids in its cell envelope (69, 70). Mce1 is thought to bridge the two membranes (53); since OmamC is predicted (HMMTop) to insert into the plasma membrane, it could interact with the Mce1 complex at this inner membrane. Such complexation would be consistent with known interactions between the Mce1 complex and other Omam proteins such as OmamA and OmamB, which reduce fatty acid uptake by Mce1 (57). Given the significant amount of cross talk between Mce proteins in the different complexes, it is possible that *mam* or *omam* genes could also be redundant in other lipid-related processes with significant overlap in their interactions and functions.

While it remains to be seen whether OmamC and Mce1 directly interact, overexpression of *omamC* either directly or indirectly enhances the uptake and utilization of fatty acids, albeit under different nutrient conditions. Fatty acid uptake primarily occurs during favorable, carbon-rich conditions, while this fat reservoir becomes a crucial energy source during carbon starvation when stored fatty acids are now utilized. The protein could do this by steering the directionality of lipid transport and functioning as a switch between uptake and utilization. Increasing the accessibility of lipids during starvation is clearly beneficial for the bacterium, although why OmamC’s selectivity for unsaturated over saturated fatty acids is advantageous for the bacterium is less clear. One possibility is that this preference might impact the biophysical properties of the membrane. For example, during starvation, OmamC’s promotion of unsaturated fatty acid consumption might leave the remaining saturated fatty acids to form a stiffer membrane. A denser, more tightly packed membrane might be less permeable to molecules the bacterium encounters in the host, such as reactive oxygen and nitrogen species, while also making it less susceptible to molecules it might encounter during antibiotic therapy, like rifampicin, as observed in bacteria overexpressing OmamC (Fig. 2C).

Interestingly, we observed OmamC-dependent effects in early dormancy (2 weeks of carbon starvation), both in observed overall higher metabolic activity in response to oleic acid (Fig. 3D; Fig. S10) and in response to THL treatment, whereas OmamC-dependent effects were not present in late dormancy (7–8 weeks of carbon starvation). These observations are consistent with previous work, which showed that the non-growing state changes over time as respiration declines (39, 71, 72). Early in dormancy, higher levels of OmamC delayed the lethal effects of THL treatment, which could be attributed to its role in generating higher concentrations of available fatty acids when THL concentration is limiting. Later in dormancy, when any surplus of lipids available via an OmamC-dependent mechanism is exhausted, OmamC would no longer provide any resistance to THL. Remarkably, THL eradicates dormant Mtb, suggesting that Mtb is dependent on lipids throughout carbon starvation, even if no longer in an OmamC-dependent manner late in starvation.

This work collectively demonstrates the importance of fatty acid metabolism for non-growing Mtb in a carbon starvation model and highlights the value of targeting proteins involved in fatty acid uptake, utilization, and biosynthesis for therapeutic intervention. Admittedly, the nutrients available to Mtb may differ *in vivo* compared with the *in vitro* conditions described herein and may even differ between *in vivo* environments (e.g., phagosome, macrophage, granuloma, or human lung during infection). In the host, Mtb resides in fatty environments (macrophages and granulomas) and therefore should have access to lipids in most stages of the infection. However, if lipids are inaccessible, it is conceivable that its own enormous lipid pool is scavenged to survive with OmamC promoting this activity. While THL was effective at eradicating dormant Mtb, it is non-specific and inhibits a wide range of lipases. Given the potential redundancy of enzymes involved in fatty acid processing in Mtb, again supporting the importance of this function, it may be necessary to inhibit a broad range of lipase activity in starving, non-growing Mtb. However, the lipases that metabolize the particularly long fatty acids unique to Mtb may be distinguishable from human lipases and thus attractive candidates for therapeutic intervention. Interfering with lipid metabolism in Mtb, in general, could render Mtb more susceptible to other antibiotics, particularly in the non-growing state, by altering membrane permeability, as may be occurring with rifampicin. Thus, Mtb’s strong dependence on lipids in dormancy could represent a vulnerability that could be exploited in the development of new antimicrobials, potentially shortening treatment strategies against TB.

## MATERIALS AND METHODS

### Bacterial strains and culture conditions

Mtb H37Rv and derivative strains were grown at 37°C in Middlebrook 7H9 (Difco) liquid culture medium supplemented with 10% oleic acid-albumin-dextrose-catalase (OADC), 0.5% glycerol, and 0.05% tyloxapol (referred to as growth in rich media in the text) or on Middlebrook 7H10 (Difco) solid culture medium supplemented with 10% OADC (BD Biosciences) and 0.5% glycerol. For carbon starvation, liquid cultures were grown to an OD_600_ of 0.8–1, washed once in carbon starvation media (Middlebrook 7H9 supplemented with 0.05% tyloxapol), and subsequently incubated in carbon starvation media at 37°C and 100 rpm. All liquid cultures had an overhead space two to four times of the culture volume for adequate aeration. Plasmid constructs carrying a P_myc1_tetO-inducible gene (e.g., *omamC*) were not induced with anhydrotetracycline (atc), unless otherwise indicated, as promoter leakage was sufficient to induce transcription.

### Cloning of deletion and overexpression constructs

For construction of overexpression plasmids, sequences were PCR amplified from genomic DNA of H37Rv and cloned into the vector pUVtetORm (73) at PacI and PstI (NEB) restriction sites. The Δ*omamC* deletion was created by recombineering as previously described (74) with few modifications. Briefly, 1 kB of flanking sequence immediately upstream of *omamC* was amplified with primers Rv1363ca-fw and Rv1363ca-rev and cloned upstream of the hygromycin resistance cassette *hyg* in the plasmid pJG1100 using *SfbI*, *PmeI*. One kilobytes of flanking sequence immediately downstream of *omamC* were amplified with primers Rv1363cb-fw and Rv1363cb-rev, and cloned downstream of the *hyg* cassette in the resulting plasmid using the *PacI* and *AscI* sites (75). The flanking regions of *omam*C were then amplified from this plasmid, along with the hygromycin cassette using primers Rv1363ca-fw and Rv1363cb-rev, and the resulting PCR product was electroporated into H37Rv harboring the plasmid-encoded Che9c RecET recombination system (pNitET-SacB-kan) (76), which facilitates replacement of genome-encoded *omamC* with the *hygromycin* cassette by allelic exchange. The mutant candidates were plated on selective media (7H9 + OADC agar containing 50 µg/mL hygromycin), and resulting colonies were tested for the insertion of the cassette using control PCR primers reading from the hygromycin cassette to a genomic region just outside the flanking regions used for the cloning. A complete list of strains, plasmids, and primers that were used in the study are listed in Tables S11 and S12.

### Construction of Mtb transposon library

Construction of the transposon library in Mtb has been described in detail (77). Briefly, *M. smegmatis* Mc^2^155 was infected at 30°C with a TM4 phage derivative carrying the conditional transposon vector phiMycoMarT7 to generate high titers of phage. TM4 is virulent at permissive temperatures (30°C) but is unable to replicate at 37°C. For the generation of the transposon pool, 10 mL of a mid-log phase Mtb (H37Rv) culture was washed with Phage buffer (50 mM Tris-HCl pH7.4, 10 mM MgSO_4_, 2 mM CaCl_2_, 150 mM NaCl) and subsequently incubated in Phage buffer for 24 hours at 37°C, concentrated 10-fold (OD_600_ = 10), and infected with the phage-carrying phiMycoMarT7 at an MOI of 10 for 3 hours at 37°C. The mix of Mtb and Phage was then cultured for 24 hours in 7H9 OADC and then pelleted and plated to 7H10 agar supplemented with 100 µg/mL kanamycin.

### Sequencing and analysis of Mtb transposon library

A volume of 10 mL dense (OD_600_ >1) Mtb culture was harvested at 3,000 rcf (Allegra X15R Centrifuge, Beckman) and resuspended in 450 µL TE-Glucose containing 10 mg lysozyme and incubated for 1 hour. To further lyse the cells, 100 µL 10% SDS was added and gently mixed. An amount of 10 mg proteinase K was then added and incubated for 30 minutes at 55°C. Finally, 200 µL 5 M NaCl and 160 µL centrimide were added and incubated at 65°C for 10 minutes. To extract the DNA, an equal volume of chloroform:isoamyl-alcohol (24:1) was added to the lysed cells, mixed, and phases were separated by centrifugation >5,000 rcf. The aqueous phase containing the DNA was precipitated using an equal volume of isopropanol, and the resulting DNA pellet was solubilized in water.

One microgram of DNA was then sheared (Covaris sonicator E220) resulting in fragments of approximately 500 bp. The sheared DNA was cleaned using AMPure XP beads (Beckman Coulter), end repaired (END-It, Epicentre) and adenosine residues were attached by Taq polymerase using 20 mM dATP at 72°C for 45 minutes (Qiagen) to facilitate adaptor ligation. The single-stranded adapters 5′-TACCACGACCA-NH_2_ and 5′-ATGATGGCCGGTGGATTTGTGNNANNANNNTGGTCGTGGTAT-3′ were denatured at 95°C and then slowly annealed using a 1% ramp down to 20°C (2 hours). This double-stranded adapter was then ligated to the A-tailed DNA fragments (T4 DNA ligase) (NEB). Adapter-ligated fragments were then purified using AMPure XP beads. Using the selective primer pairs 5′-TATGATGGGCGGTGGATTTGTG-3′ and 5′-TAATACGACTCACTATAGGGTCTAGAG-3, the region between the transposon internal T7 promoter and the adaptor was amplified using Phusion high-fidelity polymerase (NEB). To multiplex different samples for Illumina sequencing on MiSeq (Illumina), these fragments were uniquely barcoded using the primer mixes listed in Table S2. Sequences were trimmed and mapped using the software Bowtie2 (http://bowtie-bio.sourceforge.net/bowtie2/manual.shtml) and the H37Rv reference sequence (NC_000962). For the further analysis, we used the con-ARTIST script [MATLAB (Mathworks)] (47).

### GSEA analysis

Genes identified from the Tn-seq analysis having a significance value of *P* < 0.03 (Mann-Whitney *U* test) between input and output pools (Tables S4 and S5) were analyzed in the gene set enrichment analysis using KEGG gene ontology terms. Default parameters of the software GSEA (GSEA v4.0.1, Broad Institute and University of California) were used to determine the normalized enrichment score. For a better visualization, the enriched gene sets were further combined into four broader customized categories metabolism, respiration, stress/signaling, and transporter subsets. Inclusion gene set size was set very lenient to 2 as the input data set size was small (Fig. S4; Table S13).

### Transcriptome analysis

Mtb cultures were pelleted and resuspended in TRIzol (Thermo Fisher Scientific) and frozen at −80°C to preserve RNA. In general, for exponentially growing cultures (OD_600_ = 0.6–1.0), 1 mL of culture was harvested for RNA isolation. For starved cultures whose mRNA levels were substantially lower and which also contained different degrees of dead cells, we harvested 10 mL of culture. The Pellet-TRIzol mix was then bead beaten (0.1 mm beads zirconia/silica) (Biospec) three times for 1 minute with 1-minute intervals on ice to cool the sample. The lysed sample was then applied to a direct-zol RNA Miniprep column according to supplier’s protocol (Zymo Research). A range of 0.1–0.5 ug total RNA was used for library construction, described previously in detail (78). Briefly, samples were DNase treated and fragmented for barcoded adaptor ligation and sample identification. Multiple samples were then pooled, and rRNA was depleted (Ribo-Zero) (Illumina). The sequences of the adapters were then used for cDNA synthesis. After degradation of the RNA strand and a second-adaptor ligation to be able to further multiplex the libraries, we amplified these fragments with index primers for Illumina sequencing (Next-Seq 500, 800 M paired-end reads). Analysis was performed using DESeq 2.

### [1-^14^C] labeling experiments

Mtb H37Rv and derivative strains were grown to an OD_600_ of 0.8 in Middlebrook 7H9 10% OADC, 0.5% glycerol, and 0.05% tyloxapol. Then, [1-^14^C]-acetate (Perkin Elmer) was added at a concentration of 1 uC/mL and incubated for a further 12 hours. Then cultures were harvested and washed in starvation media before being resuspended in starvation media and incubated with shaking at 37°C. One day after the switch to starvation media, we sampled at *t* = 0 and after 20 days (*t* = 20). The relatively early time points were chosen to avoid measuring dead bacteria that occur later in starvation. Samples were spun down and washed in H_2_O and resuspended in H_2_O and heat killed at 80°C overnight. Preparation of the samples for thin layer chromatography has been previously described (79) with minor modifications. Briefly, saponification was performed by adding 40% tetrabutylammonium hydroxide to the samples and incubating for 20 hours at 100°C (vol/vol 1:1). Methylene chloride (vol/vol 1:1) and methyl iodide (vol/vol 1:40) were then added to the cooled mixture methylation and slowly rotated for 1 hour. After phase separation, the upper aqueous phase was discarded. This was repeated after adding 3 N HCl (vol/vol 1:2) and again repeated after adding H_2_O (vol/vol 1:2). Residual water was removed by NaSO_4_. The lipophilic phase was dried and resolved in methylene chloride and applied to a Silica Gel 60 F_254_ plate (Millipore Sigma). Separation was achieved using solvent (vol/vol) 95:5 hexane/ethyl acetate in a TLC chamber.

### Lipidome analysis

Mtb was kept in starvation media (Middlebrook 7H9 supplemented with 0.05% tyloxapol) for 3 weeks as described above with an OD_600_ = 1 at start of starvation. A volume of 3 mL of the starved culture was then pelleted at 10,000 rpm at room temperature (Eppendorf Centrifuge 5430) and resuspended in 500 µL isopropanol for lipid analysis or methanol for free fatty acids liquid-liquid extraction and 20% chloroform to ensure killing of the bacteria. To break up the hardy cell wall, silicate beads were added to the suspension, and bacteria were lysed using a Mini-Beadbeater (BioSpec). Silica beads were pelleted by centrifugation (10,000 rpm) at room temperature (Eppendorf Centrifuge 5430), and the isopropanol chloroform fraction from the resulting supernatant was allowed to evaporate to prepare the sample for LC-MS analysis. Polar and non-polar lipids were profiled using LC-MS system comprising Shimadzu Nexera X2 U-HPLC (Shimadzu Corp.) coupled to an Q Exactive HF orbitrap mass spectrometer (Thermo Fisher Scientific). Dried samples were resuspended in 100 µL of (95:5 vol/vol) isopropanol/water containing 1,2-didodecanoyl-sn-glycero-3-phosphocholine (Avanti Polar Lipids) as an internal standard and centrifuged (10 minutes, 9,000 × *g*, 4°C). Supernatants (10 µL) were injected onto a 100 × 2.1 mm, 1.7 µm ACQUITY BEH C8 column (Waters). The column was eluted isocratically at 450 µL/min with 80% mobile phase A (95:5:0.1 vol/vol/vol 10 mM ammonium acetate/methanol/formic acid) for 1 minute followed by a linear gradient to 80% mobile phase B (99.9:0.1 vol/vol methanol/formic acid) over 2 minutes, a linear gradient to 100% mobile phase B over 7 minutes, then 3 minutes at 100% mobile phase B. MS analyses were carried out using electrospray ionization in the positive ion mode using full-scan analysis over 220–1,100 m/z at 120,000 resolution and 3 Hz data acquisition rate. Other MS settings were sheath gas 50, in source CID 5 eV, sweep gas 5, spray voltage 3 kV, capillary temperature 300°C, S-lens RF 60, heater temperature 300°C, microscans 1, automatic gain control target 1e6, and maximum ion time 125 ms. Raw data were processed using Progenesis QI (nonlinear dynamics) for peak detection and integration of both lipids of known identify and unknowns. Lipid identities were determined based on comparison to reference plasma membrane extracts and are denoted by total number of carbons in the lipid acyl chain(s) and total number of double bonds in the lipid acyl chain(s). Free fatty acids were profiled using a Shimadzu Nexera X2 U-HPLC (Shimadzu Corp.) coupled to a Q Exactive hybrid quadrupole orbitrap mass spectrometer (Thermo Fisher Scientific). Dried samples were resuspended in 120 µL of 3:1 methanol water containing 15R-15-methyl-PGA_2_, 15R-15-methyl-PGF_2alpha_, 15S-15-methyl-PGD_2_, 15S-15-methyl-PGE_1_, and 15S-15-methyl-PGE_2_ (Cayman Chemical Co.) internal standards and centrifuged (10 minutes, 9,000 × *g*, 4°C). Supernatants (10 µL) were injected onto a 150 × 2 mm ACQUITY BEH C18 column (Waters). The column was eluted isocratically at a flow rate of 450 µL/min with 20% mobile phase A (0.1% formic acid in water) for 3 minutes followed by a linear gradient to 100% mobile phase B (acetonitrile with 0.1% acetic acid) over 12 minutes and then isocratic elution using 100% mobile phase B for 3 minutes. MS analyses were carried out in the negative ion mode using electrospray ionization, fullscan MS acquisition over 70–850 m/z, and a resolution setting of 70,000. Other MS settings were sheath gas 45, sweep gas 10, spray voltage −3.5 kV, capillary temperature 320°C, S-lens RF 60, heater temperature 300°C, microscans 1, automatic gain control target 1e6, and maximum ion time 250 ms. Raw data were processed using Progenesis QI (nonlinear dynamics) for peak detection and integration. Identities of free fatty acids were confirmed using authentic reference standards.

### Resazurin assay

Mtb H37Rv strains were starved at an OD_600_ of 1 for 2 or 7 weeks using the standard starvation media before it was diluted 10-fold in fresh starvation media containing 0.8 mg/mL oleic and 0.1 mg/mL resazurin. Absorbance was measured at 570 and 600 nm. Reduction grade for each time point was calculated using the following equation:


$$reduction\;grade=\;\frac{\left(\varepsilon_{OX_{600nm}}\times A_{570nm_{tx}}\right)-\left(\varepsilon_{OX_{570nm}}\times A_{600nm_{tx}}\right)}{\left(\varepsilon_{RED_{570nm}}\times A_{600nm_{t0}}\right)\;-\left(\varepsilon_{RED_{600nm}}\times A_{570nm_{t0}}\right)}$$

where ε is the molar extinction coefficient for resazurin depending on the wavelength and its oxidation state, *A* is the absorbance at a given wavelength, *t*_0_ is the initial measurement, and *t*_*x*_ is the measurement at a given time *x*. The values for the extinction coefficient are the following: εOX__600nm_ = 117.216, εOX__570nm_ = 80.586, εRED__570nm_ = 155.677, and εRED__600nm_ = 14.652 [Biosystems, (80)]. The experiments were performed in sextuplicate in black 96 well plates with clear flat bottom (Corning) in a final volume of 100 µL per well. The microtiter plates were incubated in a closed container with a damp towel at 37°C.

### Macrophage infection model

Infections were carried out as previously described (81). Wild-type Mtb H37Rv, H37RvΔ*omamC*, or H37Rv harboring the *omamC* overexpression plasmid was used to infect J774 macrophages (ATCC). Mtb strains used were grown to mid-log phase, washed in PBS, resuspended in PBS, and subjected to a low-speed spin to pellet clumps. J774 were infected at the indicated MOI, allowing 4 hours for phagocytosis. Cells were then washed once with PBS, and media were added back to washed, infected cells. Infected J774 were incubated for 3 days, then macrophages were lysed with Triton X-100 (0.5%), and surviving bacteria were enumerated by colony-forming units. Cell lines were verified to be free of mycoplasma contamination using the ATCC Universal Mycoplasma Detection Kit.

### THL killing assay

Bacterial cultures were exposed to carbon starvation as described in Bacterial strains and culture conditions. In this case, bacteria were kept in 2 mL starvation media in a tightly sealed 30 mL flask gently shaken at 100 rpm. To investigate the effects of lipase inactivation, 30 µg/mL tetrahydrolipstatin (Orlistat, Sigma) was added to the media early on starting at week 1 and re-applied at weeks 2 and 3. For the effects in the late stage of carbon starvation, THL was added at weeks 7, 8, and 9. Each week, the cultures were sampled, and dilutions plated on Middlebrook 7H10 (Difco) solid culture medium supplemented with 10% OADC (BD Biosciences) and 0.5% glycerol.

### THL inhibition of mycolyltransferase activity

Inhibition of mycolyltransferase activity by THL in *M. tuberculosis* H37Rv was examined using an optimized quencher-trehalose-fluorophore (N-QTF) (82). Bacteria were grown to OD_600_ = 0.2, and mycolyl transferase activity was measured by adding N-QTF (2.5 µM) in a final volume of 50 µL per well (*n* = 3). Relative fluorescence units were measured at ex/em 485/525 and monitored for 80 hours at 37°C. Each triplicate per strain is shown together with a linear fit (sampling frequency 1 /hour, all *R*2 >0.98, rates: wt = 13.4 h^−1^, corrected for cell number).

## SUPPLEMENTAL MATERIAL

Supplemental Figures mbio.03208-23-s0001.pdf Figures 1-17.

Supplemental Tables mbio.03208-23-s0002.xlsx Tables 1-13.

ASM does not own the copyrights to Supplemental Material that may be linked to, or accessed through, an article. The authors have granted ASM a non-exclusive, world-wide license to publish the Supplemental Material files. Please contact the corresponding author directly for reuse.
