# Supplementary material for: Genetic factors affecting storage and utilization of lipids during dormancy in Mycobacterium tuberculosis
Source: mBio. 2024 Jan 18;15(2):e03208-23. doi: 10.1128/mbio.03208-23 (PMC10865790; doi:10.1128/mbio.03208-23)
Supplement: Supplemental Figures — Figures 1-17. [file mbio.03208-23-s0001.pdf]

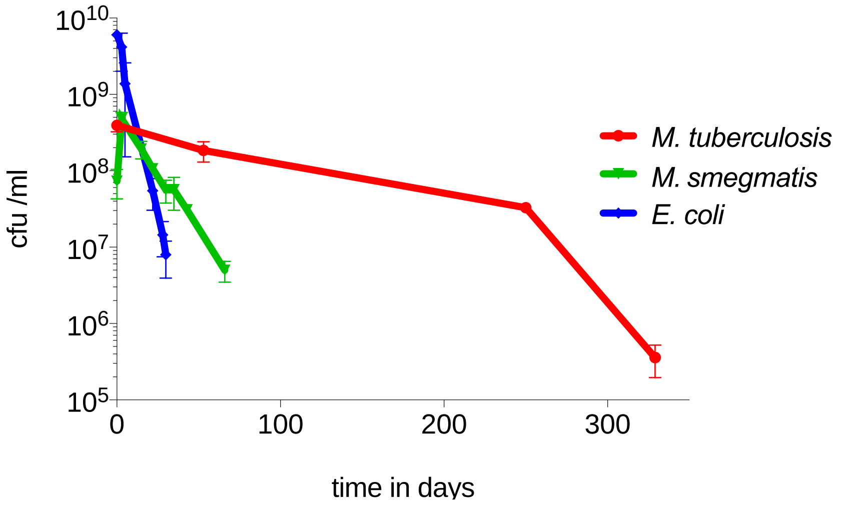

Suppl. Figure 1 Survival of *E. coli* K12, *M. smegmatis* Mc^{2}155 and *M. tuberculosis* H37Rv in carbon-starvation media. A late logarithmic culture of *E. coli* was grown in M9 media containing 0.4 % glucose and then washed in M9 salts and starved in M9 salts. *M. smegmatis* and *M. tuberculosis* were grown to late logarithmic phase in Middlebrook 7H9 (10 % OADC, 0.5 % glycerol, 0.05 % Tyloxapol) and then washed and kept in Middlebrook 7H9 (0.05 % Tyloxapol). We sampled at the indicated time points and plated for cfus on either LB (*E. coli*) or Middlebrook 7H10 (10 % OADC, 0.5 % glycerol) plates for *Mycobacteria*.

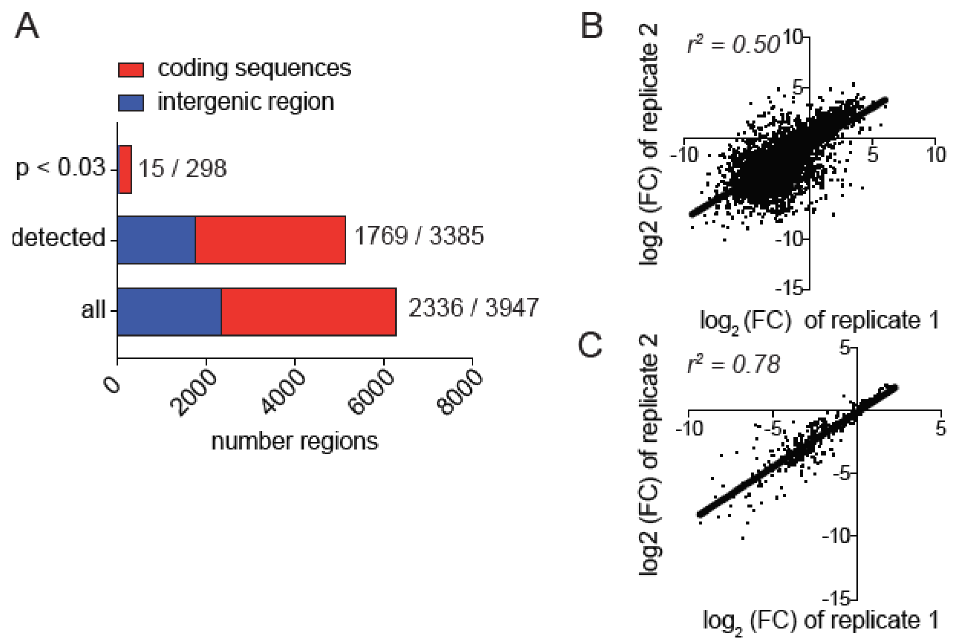

Suppl. Figure 2 Transposon insertion sequencing of carbon-starved H37Rv (compare Figure 1 for more details). **A)** Using the Con-Artist program we detected 1769 of 2336 intergenic regions and 3385 of 3947 annotated genes in H37Rv of which 15 intergenic regions and 298 genes had a $p < 0.03$. Eventually, 6 intergenic regions and 102 genes were defined as hits with a log2 (FC) of < 3.5 between input and output pool. **B and C)** We worked with two biological replicates that were sequenced and afterwards both starved for five weeks in Middlebrook 7H9 (0.05 % Tyloxapol) and afterwards resuscitated in Middlebrook 7H9 (10 % OADC, 0.5 % glycerol, 0.05 % Tyloxapol). We repeated this twice and sequenced the transposon mutant pool after this again. Both pools were compared with the Con-Artist program to check for conditional essentiality. The resulting log2 (FC) that showed a $p < 0.03$ correlated with an $R^2 = 0.78$. In B) the log2(FC) of all detected genes and intergenic regions are shown while C) depicts genes and intergenic regions with a $p < 0.03$.

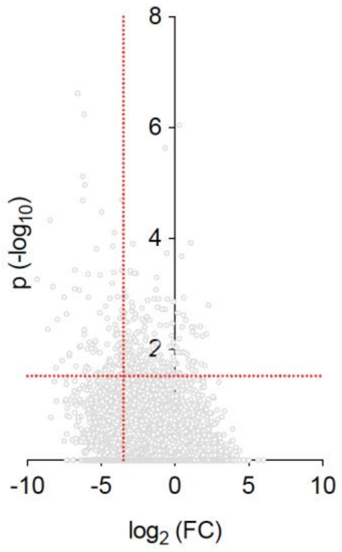

Suppl. Fig. 3 Results from the transposon screen using transposon insertion sequencing and the Con-Artist pipeline for analysis. The changes between original transposon mutant pool and the transposon mutant pool after three cycles of growth, starvation and resuscitation are depicted. We chose a log_2 (FC) cut-off of - 3.5 (vertical red dotted line) and a p-value < 0.03 ($-log_{10}$ > 1.52, horizontal red dotted line) leading to 102 genes defined as hits. In total 3385 genes were detected (Suppl. Table 5).

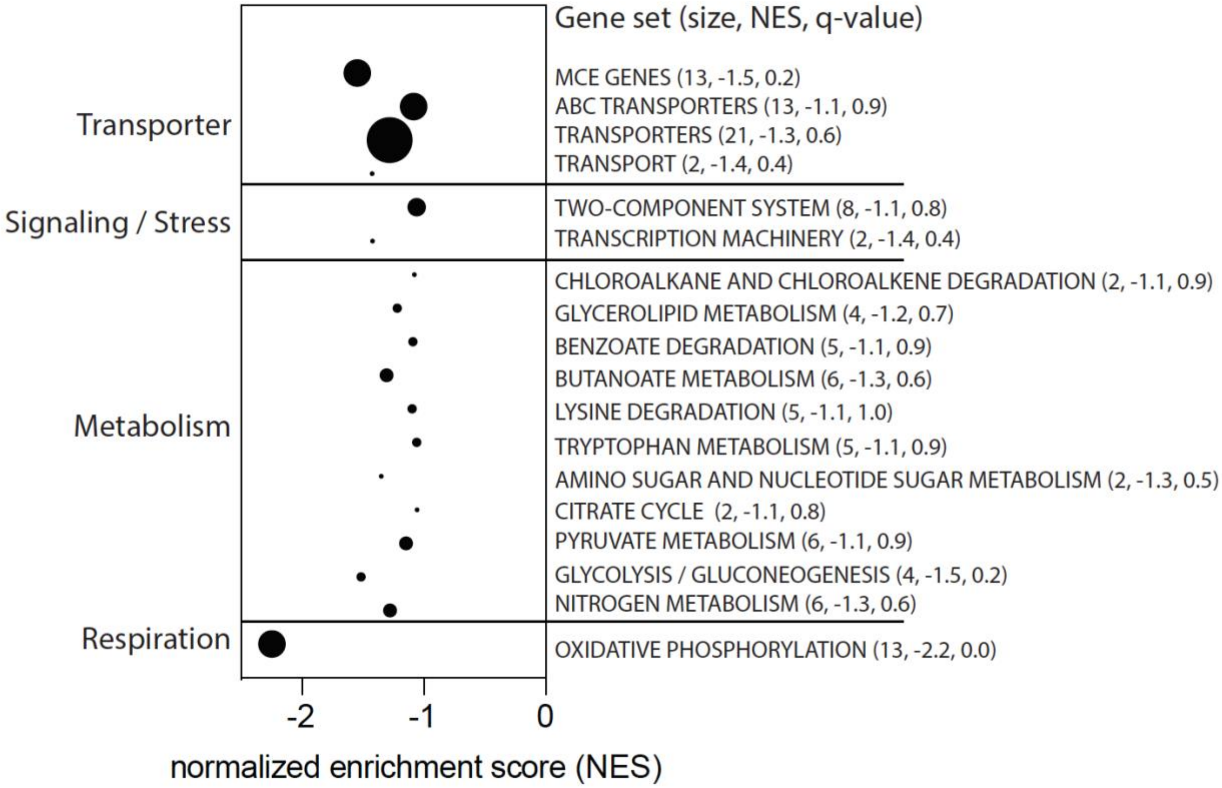

Suppl. Figure 4 GSEA Analysis. Hits from the Tn-seq analysis with a $p < 0.03$ were analyzed using gene set enrichment analysis to find pathways overrepresented in the dataset (GSEA v4.0.1, Broad Institute and University of California). We used KEGG gene ontology terms. Sizes of circles represent the number of genes of the enriched gene set, bigger circles represent bigger gene sets. Higher order terms: transporter, signaling/.stress, metabolism and respiration were chosen by us and used to combine different gene sets in even broader terms mainly for visualization purposes in Figure 1B.

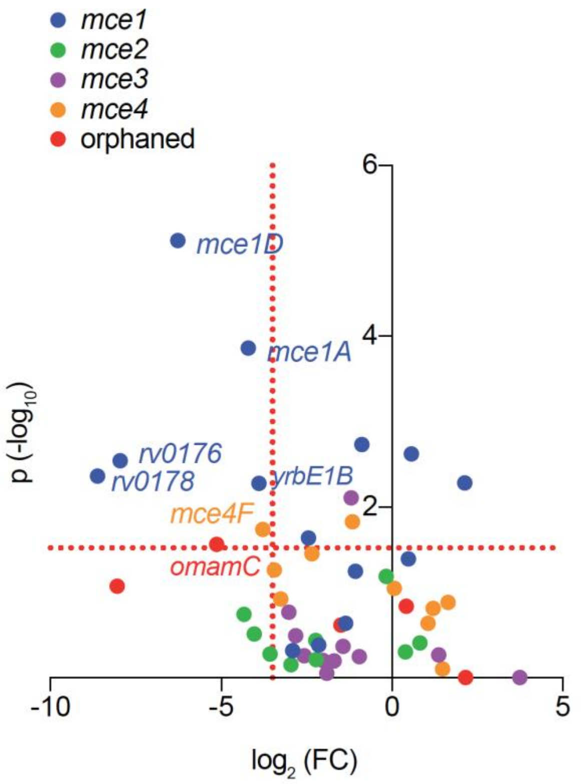

Suppl. Figure 5 ***Mce*** **genes of H37Rv** highlighted from (Fig. 1B), mce clusters one to four and the uncharacterized genes *omamC* and *omamD* outside the *mce* clusters that share homology to *mce* associated genes, in particular *rv0177* and *rv1972* (Suppl. Fig. 9). Hits (log_2 (FC) < 0.03 and ($-log_{10}$) p > 1.52) include from the *mce1* cluster: *yrbE1B (rv0168*), *mce1E (rv0169)*, *mce1D (rv0172*), and the *mce1* associated genes *rv0176*, *rv0178*; *mce4* cluster: *mce4F* (*rv3494c)*; orphaned *mce* associated: *omamC* (*rv1363c)*

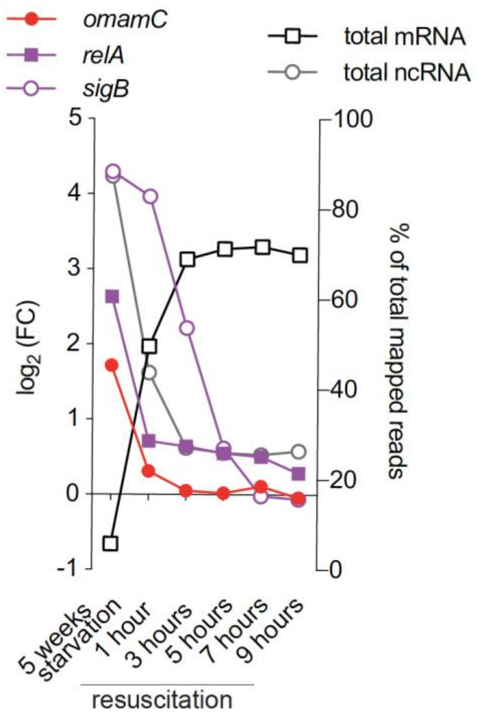

Suppl. Figure 6 Differential expression analysis. Relative mRNA levels (indicated time point on x-axis vs logarithmic growth, DESeq2) in wt H37Rv. mRNA levels of *omamC* and both stress regulators *sigB*, *relA* are similarly falling during resuscitation (left y-axis). The ratio between ncRNA and mRNA pools changes over time too. We found a very high relative abundance of ncRNA (>80 %) and a very low abundance of mRNA (<10 %, discrepancy through left over rRNA after rRNA depletion for library construction) during starvation for (i) gene transcription is lower during starvation as expected during dormancy, (ii) mRNAs are generally longer than ncRNA and are more prone to be degraded. As expected, as soon as H37Rv was exposed to nutrient rich and growth-permissive conditions, the mRNA levels rise abruptly (right y-axis).

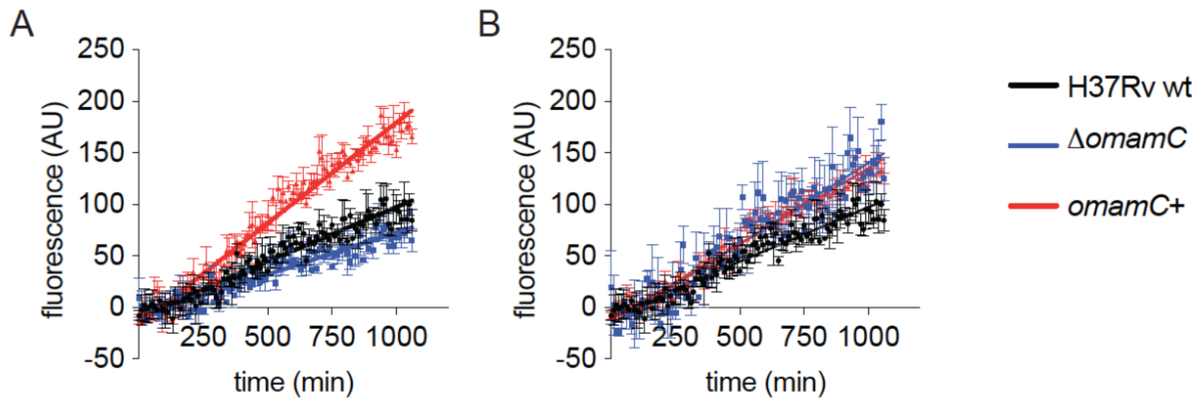

Suppl. Figure 7 Viability and permeability stain of carbon-starved H37Rv. 5-weeks starved cultures in starvation media (OD_{600} = 0.2) were incubated with 1 mM non-fluorescent Calcein AM that after penetrating the mycobacterial cell wall of living cells is cleaved into fluorescent calcein (λ_{ex} 496 nm; λ_{em} 516 nm). It can thus be used to measure for cell viability (A). After 5 weeks, there were more *omamC*+ alive than wt and Δ*omamC* (k_{wt} = 0.11, $k_{\Delta omamC}$ = 0.08, k_{omamC+} = 0.20). (B) However, when we normalized the fluorescence values by cfu, fluorescence values overlap almost identically suggesting that there is no difference in permeability per cell (k_{wt} = 0.11, $k_{\Delta omamC}$ = 0.15, k_{omamC+} = 0.14, cfu_{wt} = 8.7E+06 +/- 1.0E+06 ml^{-1}, $cfu_{\Delta omamC}$ = 4.5E+06 +/- 1.3E+06 ml^{-1}, cfu_{omamC+} = 1.2E+07 +/- 2.6E+06 ml^{-1}). Experiment was performed in triplicates, depicted is the mean and error bars represent standard deviation.

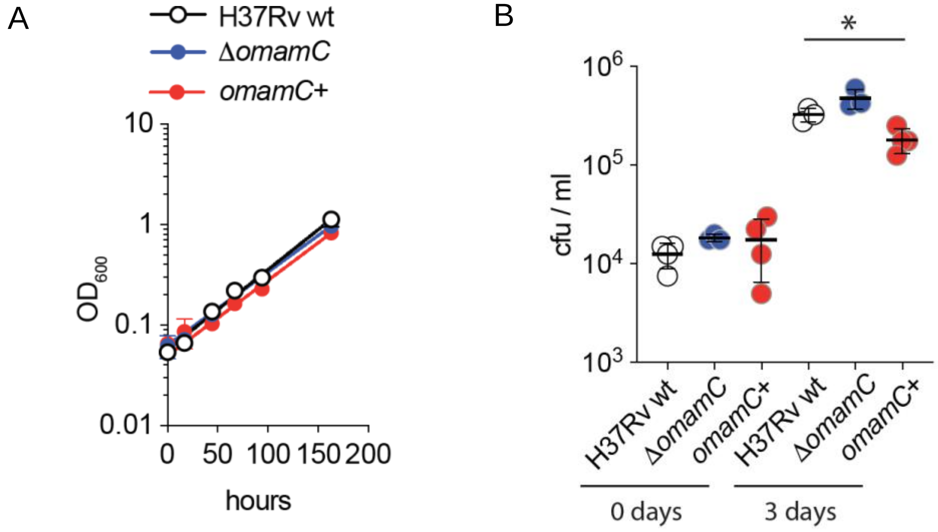

Suppl. Fig. 8 Infection of J774 A) Growth rate of these strains determined by OD600 in growth media (7H9 salts, 10% OADC, 0.5 % Glycerol, 0.05 % Tyloxapol). B) J774 macrophages were infected with H37Rv wild type, omamC or omamC+ and incubated for 3 days. Initial and final number of intracellular bacteria was determined by cfu, black line and error bars depict mean and standard deviation, at 3 days omamC+ = 0.01.

```
Rv0177       ----------------------------------------------------MSPRR---   5
Rv1363c      MAETTEPPSDAGTSQADAMALAAEAEAAEAEALAAAARARARAARLKREALAMAPAEDEN   60
Rv1972       ----------------------------------------------------MSVAV---   5
                                                                 *:

Rv0177       ------------KFEPG--------EGALLAPQSIEPSRRWGLPLALTASAVVMAAAISA   45
Rv1363c      VPEEYADWEDAEDYDDYDDYEAADQEAARSASWRRRLRVRLP----RLSTIAMAAAVVII   116
Rv1972       ------------DSDAEDDAVSEIAEAAGVSPAPAKPSMSAPRRMLLFGLVVVVALAVLL   53
                         . :          *.*  :    .            .  .: * .:

Rv0177       CALMRISHE---SHQRAA---HKDIVMLSDVRSFMTMFTSPDPFHANEYAERVLSHATGD   99
Rv1363c      CGFTGLSGYIVWQHHEATERQQRAAAFAAGAKQGVINMTSLDFNKAKEDVARVIDSSTGE   176
Rv1972       CCW----GF---RVQRARHAQDQRGHFLQAARQCALNLTTIDWRNAEADVRRILDGATGE   106
             *             :.*    .:   :   .:.    :*: *  :*:  . *::. :**:

Rv0177       FAKQYHERANDILIRISG-VEPTTGTVLDAGVQRWNEDGSANVLVVTQITSKSADGKRVV   158
Rv1363c      FRDDFQQRAADFTKVVEQSKVVTEGTVNATAVESMNEHSAVVLVAATSRVT-NSAGAKDE   235
Rv1972       FYNDFAQRSQPFVEVLRHAKASTVGTITEAGLQTQTADTAQALVAVSVQTS-NAGEADPV   165
             * .:: :*:  :   :      * **:  :.::  . . :  ::..:  .:  .:

Rv0177       SNANRWLVTAKQEGNEWKISSLLPVI  184
Rv1363c      PRAWRLKVTVTEEGGQYKMSKVEFVP  261
Rv1972       PRAWRMRITVQRVGDRVKVSDVGFVP  191
              .* *  :*. . *.. *:*.:  *
```

Suppl. Figure 9 Sequence alignment of the orphaned mce associated gene *omamC* (*rv1363c)* versus *rv0177* (*mce* cluster 1) and *rv1972* (*mce* cluster 3) using CLUSTAL O (1.2.4)

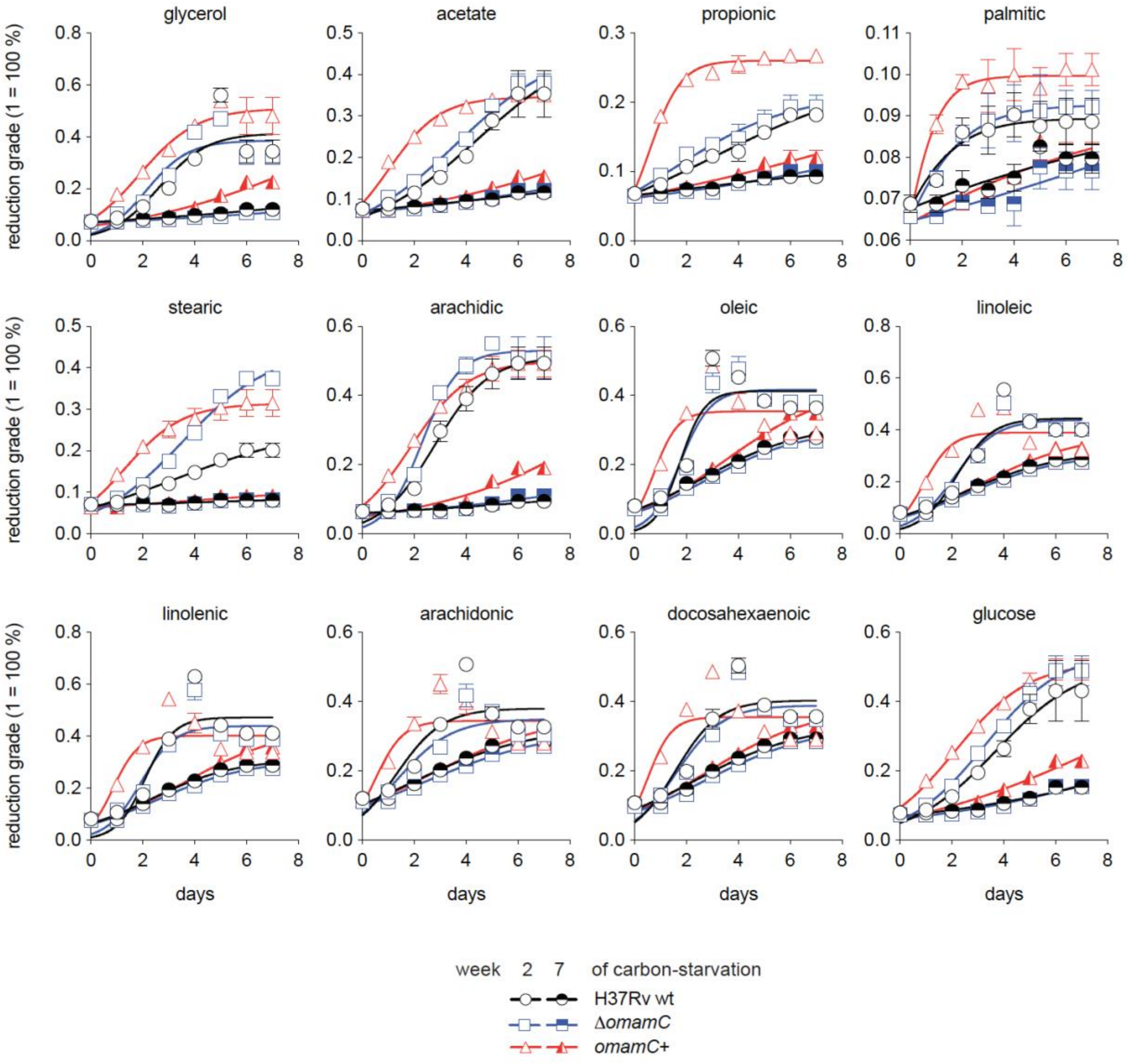

Suppl. Figure 10 Consumption of different fatty acids after 2 and 7 weeks of carbon-starvation. The redox sensitive dye resazurin allows to measure the intensity and time it takes H37Rv, Δ*omamC*, *omamC*+ to metabolize the indicated glycerol, glucose and fatty acids. For that purpose we measured absorbance A_{600nm} and A_{570nm} and calculated the reduction grade (1 = 100 %, $((\varepsilon_{OX\_600nm} * A_{570nm\_tx} - \varepsilon_{OX\_570nm} * A_{600nm\_tx}) / (\varepsilon_{RED\_570nm} * A_{600nm\_t0} - \varepsilon_{RED\_600nm} * A_{570nm\_t0})$; ε_{OX_600nm} = 117'216, ε_{OX_570nm} = 80'586, ε_{RED_570nm} = 155'677, ε_{RED_600nm} = 14'652). Every timepoint was measured in sextuplicates, shown is the mean and standard deviation. The time points are fitted in a logistic growth curve.

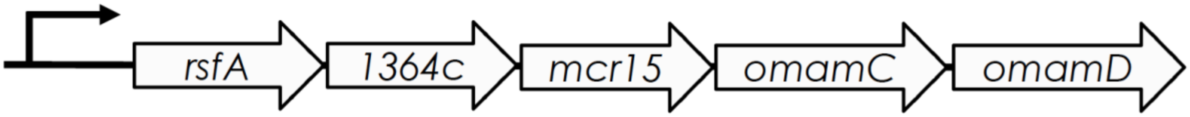

Suppl. Figure 11 The annotated *rsfA* operon entails putative stress regulators *rsfA* and *rv1364c*, a non-coding RNA *mcr15* and the two putative orphaned *mce* associated genes *omamD* and *omamC*.

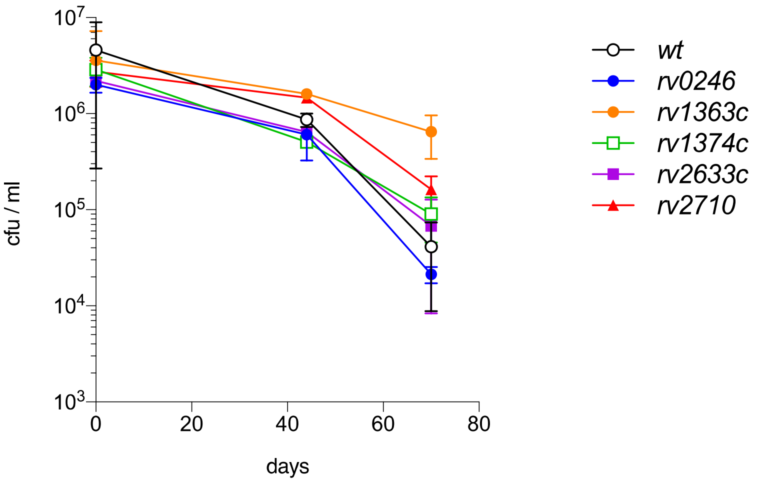

Suppl. Figure 12 Survival rates of reference genes for determining important differential gene expression patterns in *omamC+* (Compare Fig. 4A). Disruptions of these genes were hits in the Tn-seq screen but their over-expressions did not result in a substantial benefit comparable to *omamC+*. Cfus were determined by plating on 7H10 plates (10 % OADC, 0.5 % glycerol) at 37 °C. Depicted is the mean and standard deviation.

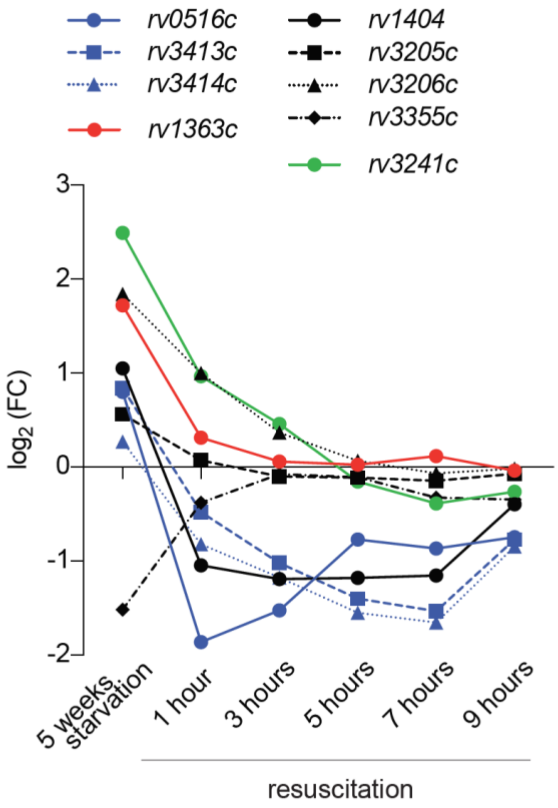

Suppl. Figure 13 Gene expression in Fig. 4A during carbon starvation and early resuscitation. mRNA levels of H37Rv under five weeks carbon-starvation and early resuscitation were determined by RNAtag-seq and analyzed by DESeq2 (R). All $\log_2$(FC) values are compared to exponentially growing H37Rv wt.

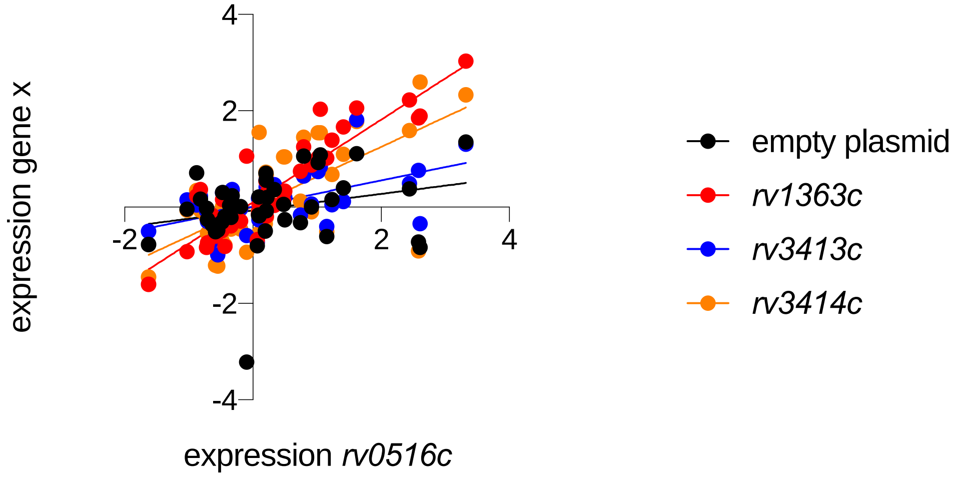

empty plasmid	*rv1363c*	*rv3413c*	*rv3414c*
0.06129	0.8178	0.2939	0.4418

Supp. Figure 14 Expression of *sigD* regulon genes (Raman et al., 2004) in a *rv0516c*+ (α-α-*sigD*) correlated to *omamC*+ (*rv1363c*, R^2 = 0.81), but correlated poorly with *sigD* (*rv3414c*, R^2 = 0.44) and α-*sigD* (*rv3413c*, R^2 = 0.29) and no correlation with pUV15tetORm. The genes within the *sigD* regulon are listed in Suppl. Table 13.

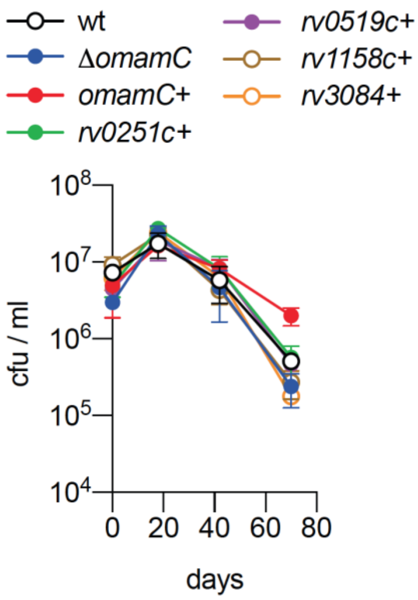

Supp. Figure 15 CFU based survival assay of hits from Fig. 4E. MTB strains were grown to an OD600 = 1 and exposed to carbon starvation methods as described in Materials and and Methods and identical to Figure 2A. At indicated timepoints the carbon straved cultures were sampled and plated on 7H9 + OADC agar plates and incubated for three weeks to assess the number of colonies.

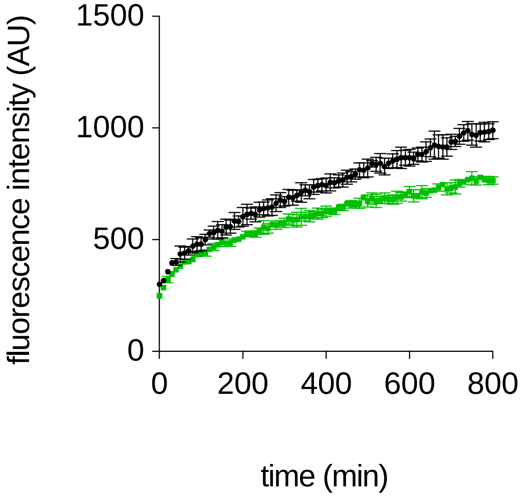

Suppl. Figure 16 Inhibition of Ag85 by THL in H37Rv. 2.5 μM of the probe N-QTF were added to an exponentially growing culture of H37Rv in the absence (black) or presence (green) of the inhibitor THL (30 μM). Fluorescence (λ_{ex} = 480 nm, λ_{em} = 530 nm) was measured every 10 min.

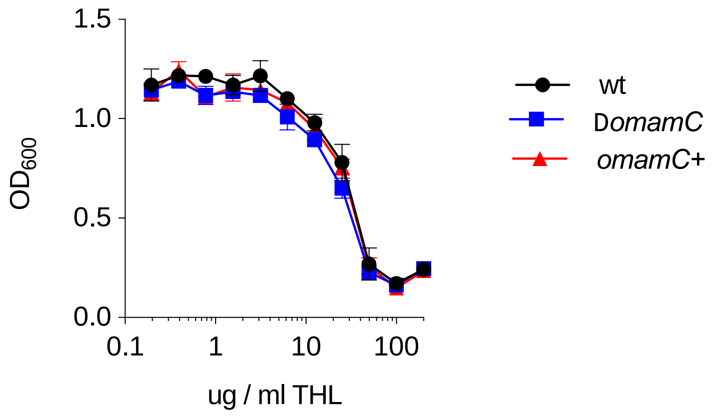

Suppl. Figure 17 MIC of Tetrahydrolipstatin (THL, Orlistat). The strains H37Rv, Δ*omamC*, *omamC* + were grown in 7H9 (10 % OADC, 0.5 % glycerol, 0.05 % tyloxapol) to late logarithmic phase and then diluted to an OD_{600} = 0.05 in same media supplemented with different concentrations of THL and incubated at 37 °C for two weeks and subsequently the OD_{600} was measured. Each sample in quadruplicates. Shown is mean and standard deviation.
